# Supplementary figures and images for: Hypothalamic corticotropin-releasing hormone neurons modulate sevoflurane anesthesia and the post-anesthesia stress responses
Source: eLife. 2024 Nov 11;12:RP90191. doi: 10.7554/eLife.90191 (PMC11554309; doi:10.7554/eLife.90191)

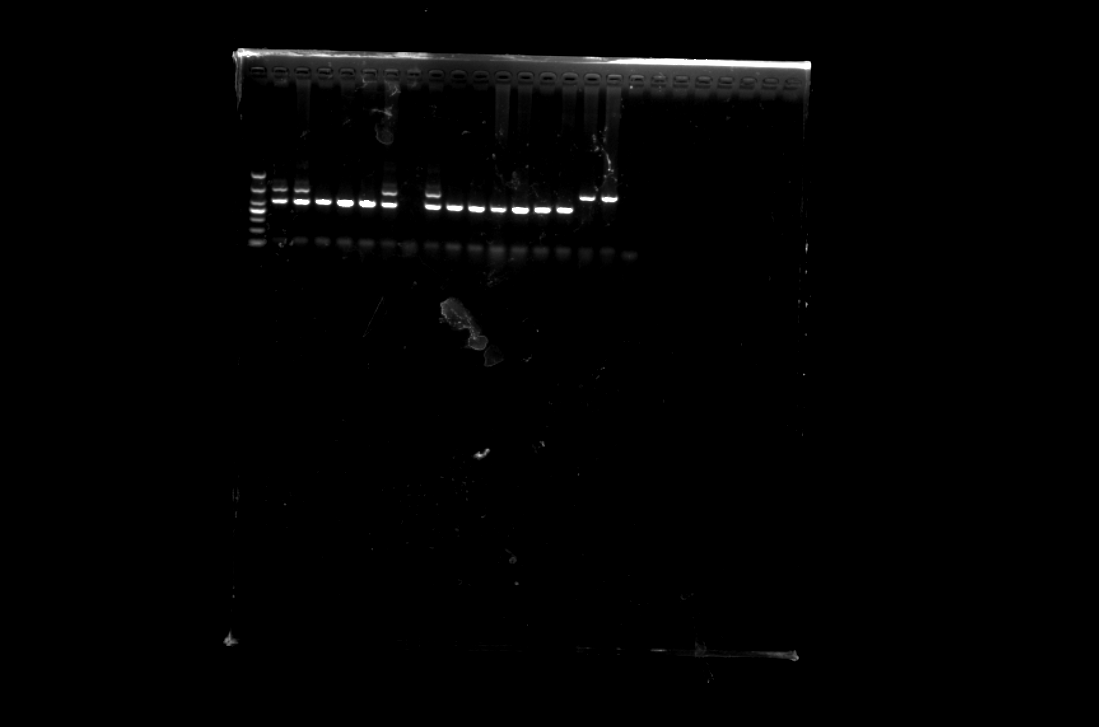

Supplement: Figure 1—figure supplement 3—source data 1. [file elife-90191-fig1-figsupp3-data1.zip › Figure 1- figure supplement 3 - source data 1/Figure 1- figure supplement 3 - source data 1.tif]

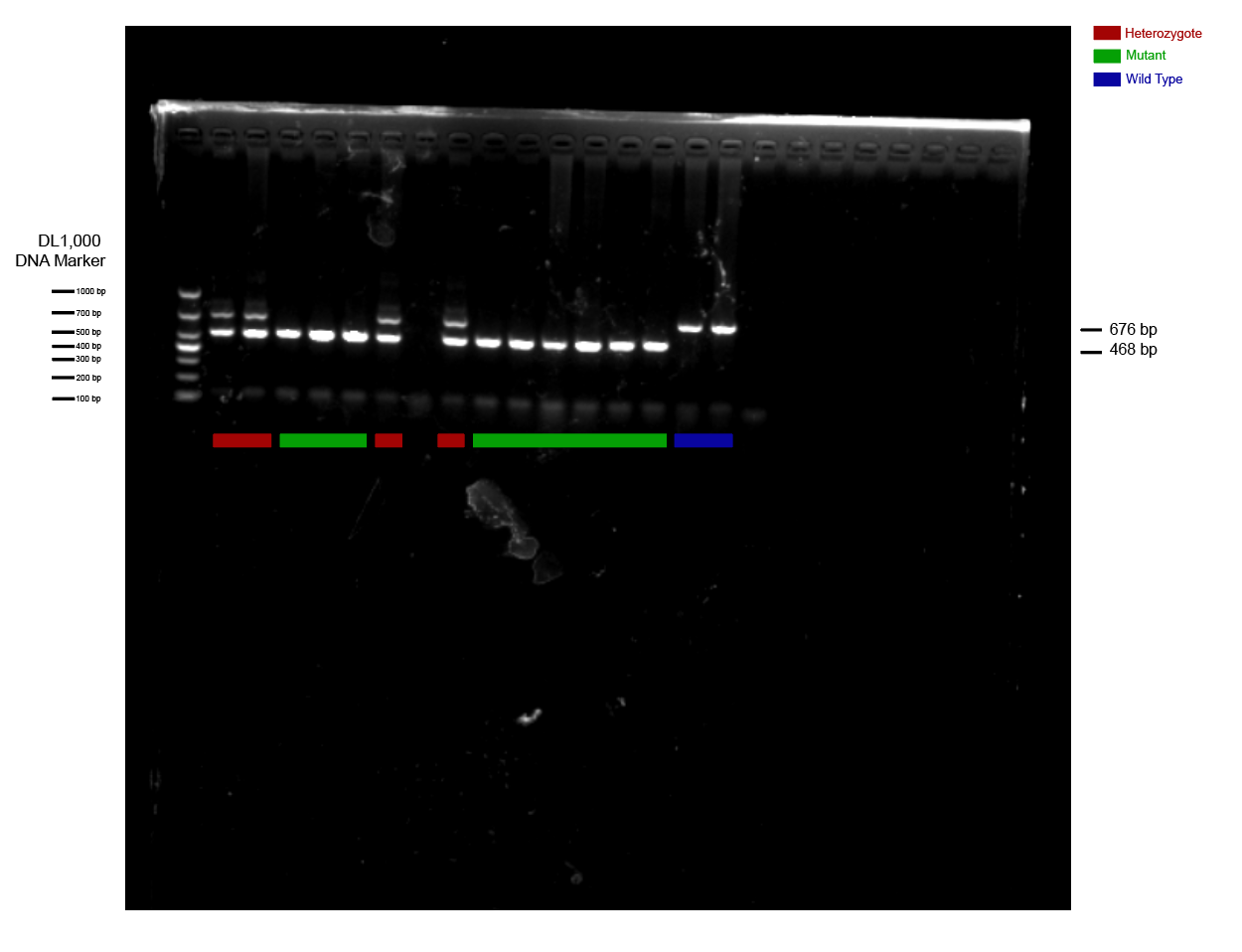

Supplement: Figure 1—figure supplement 3—source data 2. [file elife-90191-fig1-figsupp3-data2.zip › Figure 1- figure supplement 3 - source data 2/Figure 1- figure supplement 3 - source data 2.docx]
