# Supplementary material for: Hypothalamic corticotropin-releasing hormone neurons modulate sevoflurane anesthesia and the post-anesthesia stress responses
Source: eLife. 2024 Nov 11;12:RP90191. doi: 10.7554/eLife.90191 (PMC11554309; doi:10.7554/eLife.90191)
Supplement: Figure 4—source data 2. [file elife-90191-fig4-data2.docx]

Figure 4-source data 2. Mean value of EC50 for sevoflurane dose-response curves.

| EC_50_ (%) | mCherry | | Chemogenetic activation | | Chemogenetic inhibition | | Genetic ablation |
| --- | --- | --- | --- | --- | --- | --- | --- |
| LORR | | | | | | | |
| Mean | Vehicle | CNO | Vehicle | CNO | Vehicle | CNO |  |
| PVH^CRH^ neurons | 1.491 | 1.517 | 1.516 | 1.650 | 1.500 | 1.337 | 1.358 |
| 95% CI |  | | | | | | |
| PVH^CRH^ neurons | 1.444 to 1.536 | 1.292 to 1.690 | 1.509 to 1.522 | 1.616 to 1.682 | 1.496 to 1.504 | 1.297 to 1.368 | 1.292 to 1.406 |
| RORR | | | | | | | |
| Mean | Vehicle | CNO | Vehicle | CNO | Vehicle | CNO |  |
| PVH^CRH^ neurons | 1.420 | 1.571 | 1.538 | 1.791 | 1.424 | 1.306 | 1.326 |
| 95% CI |  | | | | | | |
| PVH^CRH^ neurons | 1.247 to 1.587 | 1.228 to 1.953 | 1.483 to 1.607 | 1.742 to 1.909 | 1.254 to 1.835 | 1.282 to 1.333 | 1.234 to 1.378 |

EC_50_: Alveolar concentration at which half of the mice lose their righting reflex, 95% CI: 95% confidence index.
