# Supplementary material for: Hypothalamic corticotropin-releasing hormone neurons modulate sevoflurane anesthesia and the post-anesthesia stress responses
Source: eLife. 2024 Nov 11;12:RP90191. doi: 10.7554/eLife.90191 (PMC11554309; doi:10.7554/eLife.90191)
Supplement: Figure 5—source data 1. [file elife-90191-fig5-data1.docx]

Figure 5-source data 3. Behavioral responses of CRH-Cre mice under sevoflurane steady-state GA during photostimulation.

| Group | Leg movement | Head movement | Tail  movement | Righting  reflex | Walking | Total  score |
| --- | --- | --- | --- | --- | --- | --- |
| 1-ChR2 | 2 | 2 | 2 | 2 | 2 | 10 |
| 2-ChR2 | 2 | 2 | 2 | 2 | 2 | 10 |
| 3-ChR2 | 2 | 2 | 1 | 2 | 2 | 9 |
| 4-ChR2 | 2 | 2 | 2 | 2 | 1 | 9 |
| 5-ChR2 | 2 | 2 | 2 | 2 | 0 | 8 |
| 6-ChR2 | 2 | 2 | 2 | 2 | 0 | 8 |
| 1-mCherry | 0 | 0 | 0 | 0 | 0 | 0 |
| 2-mCherry | 0 | 0 | 0 | 0 | 0 | 0 |
| 3-mCherry | 0 | 0 | 0 | 0 | 0 | 0 |
| 4-mCherry | 1 | 0 | 0 | 0 | 0 | 1 |
| 5-mCherry | 0 | 0 | 0 | 0 | 0 | 0 |
| 6-mCherry | 0 | 0 | 0 | 0 | 0 | 0 |

Leg, head, and tail movements, as well as states of righting reflex and walking in each mouse were determined during 60-s optical stimulation while mouse still inhaled with constant sevoflurane. The total score is the sum of the above categories.
